# Using GPS tracking and stable multi-isotopes for estimating habitat use and winter range in Palearctic ospreys

**DOI:** 10.1007/s00442-021-04855-5

**Published:** 2021-01-21

**Authors:** Flavio Monti, Aloїs Robert, Jean-Marie Dominici, Andrea Sforzi, Rafel Triay Bagur, Antoni Muñoz Navarro, Gaël Guillou, Olivier Duriez, Ilham Bentaleb

**Affiliations:** 1 Department of Physical Sciences, Earth and Environment, University of Siena, Via Mattioli 4, 53100 Siena, Italy; 2 CEFE, Univ Montpellier, CNRS, EPHE, IRD, Univ Paul Valéry Montpellier 3, 1919 Route de Mende, 34293 Montpellier, France; 3 Réserve Naturelle Scandola, Parc Naturel Régional de Corse, 20245 Galeria, France; 4 Maremma Natural History Museum, Strada Corsini 5, 58100 Grosseto, Italy; 5 IME (Institut Menorquí d’Estudis), Camí des Castell 28, 07702 Maó, Spain; 6 Grup Balear d’Ornitologia I Defensa de La Naturalesa (GOB), Manuel Sanchis Guarner 10, 07004 Palma de Mallorca, Spain; 7 Littoral, ENvironnement et SociétéS (LIENSS, UMR 7266), Université de La Rochelle, Bâtiment Marie Curie Avenue Michel Crépeau, 17042 La Rochelle Cedex 1, France; 8 Université Montpellier - UMR 5554, ISEM C/C 065 Place Eugène Bataillon, 34095 Montpellier cedex 05, France

**Keywords:** Feather isotope ratios, Foraging ecology, Aquatic habitat, Gps tracking, *Pandion haliaetus*, Raptor

## Abstract

We used both satellite tracking and carbon, nitrogen and sulphur stable isotopic analysis (SIA) to infer wintering ecology and habitat use of the Corsican osprey *Pandion haliaetus* population. A control sample of feathers from 75 individuals was collected within the osprey’s northern hemisphere breeding range, to assess the SIA variability across habitat types. An experimental set of SIA on feathers of 18 Corsican adults was examined to infer wintering ground locations and habitat types used during the non-breeding period. We calibrated the SIA using GPS/GSM tracks of 12 Mediterranean adults’ movements as wintering site references. We found 50% of individuals were resident and the other half migrated. Ospreys spent the winter at temperate latitudes and showed a high plasticity in habitat selection spread over the Mediterranean basin (marine bays, coastal lagoons/marshland, inland freshwater sites). Complementary to GPS tracking, SIA is, at a broad geographical scale, a reliable method to determine whether ospreys overwinter in a habitat different from that of their breeding area. This study proved that the integration of SIA and GPS/GSM tracking techniques was effective at overcoming the intrinsic limits of each method and achieving greater information for basic ecological studies of migratory birds in aquatic environments.

**Supplementary Information:**

The online version contains supplementary material available at 10.1007/s00442-021-04855-5.

## Introduction

Animal population dynamics are dependent on a series of factors, which occur during different periods of the annual cycle (Newton 2003). To study migratory birds, it is therefore mandatory to understand the ecology of the species at each biological phase (e.g., breeding, migration and wintering). For decades, migratory movements of birds have been studied by means of ring-recovery data (Baillie et al. 2009). Advances in technology and chemistry provided tools for gathering essential information on individual movements and connectivity between breeding, wintering and stopover sites (Webster et al. 2002; Trierweiler et al. 2014; Choi et al. 2020). Satellite (GPS) tracking reveals wintering grounds and the migratory routes (tracks) followed between breeding and non-breeding areas with high accuracy and precision (Phipps et al. 2019; Sarà et al. 2019). Carbon, Nitrogen, Sulphur, Deuterium and Oxygen Stable Isotope Analyses (SIA), measured in feathers or other tissues, allowed further determination of seasonal habitat use and diet (Hobson 1999; Atkinson et al. 2005; Blight et al. 2014; Hobson and Wassenaar 2018). The rationale of SIA relies on tissues like feathers, which integrate chemical elements that are ingested through the diet during the growth period (post-moult) and do not change their composition over time (Hobson 1999; Ramos et al. 2009). This technique has the advantage of being minimally intrusive and relatively cheap (Hobson 1999). Combining these two techniques allowed collecting comprehensive information at the population level, improving the understanding of marine and terrestrial populations’ year-round ecology (Bentaleb et al. 2011) a crucial cue for planning adequate management measures at the appropriate scale (Inger and Bearhop 2008; Vander Zanden et al. 2018).

Isotopes of hydrogen and oxygen are typically used to explore movement ecology of terrestrial animals because of the well-known rainwater latitudinal patterns (Bowen 2010; Hobson and Wassenaar 2018). However, these isotopes function poorly for exploring the movement ecology of marine organisms or organisms that feed principally in aquatic/marine environments, especially over reduced spatial scales, because of the relatively low range of both hydrogen and oxygen stable isotope values in marine environments (e.g., Trueman and St. John Glew 2019). This may specifically affect the study of species living in strict association with marine or coastal environments, like seabirds (Bond and Jones 2009), where high spatial resolution reference maps of the isotopic landscape are missing (i.e., isoscapes; West et al. 2010). Among the other isotopic ratios, carbon and nitrogen (δ^13^C, δ^15^N) have been more frequently used as they are acknowledged as good indicators of foraging ecology patterns in terrestrial and aquatic environments (Hobson 1999; Bentaleb et al. 2011; Ceia et al. 2015). δ^13^C of primary producers vary predictably among ocean basins, with high-latitude pelagic ecosystems having much lower δ^13^C than similar ecosystems at lower latitudes (e.g.,Bentaleb et al. 1998; Cherel and Hobson 2007; Authier et al. 2012). δ^15^N of primary producers reflects the nitrogen cycle and the nitrate demand, varying with respect to marine productivity (e.g., Goericke and Fry 1994; Altabet et al. 1999). Knowing the baseline nitrogen isotopic value allow theoretically the prediction of the δ^15^N signatures of components of a specific trophic chain applying an N isotopic fractionation of 3.4 per mil (DeNiro and Epstein 1971). However, different processes such as ontogenetic developments for mammals (e.g., milk consumption; Authier et al. 2011) need to be assessed in some cases. More recent studies in ecology show an increasing interest on Sulphur isotopic ratios (δ^34^S) as they represent an efficient tool to discriminate between freshwater and coastal/marine habitats (Hobson 1999; Knoff 2000; Caccamise et al. 2000; Lott et al. 2003). Even though this approach does not provide exact determination of wintering ground location, it allows evaluating patterns of habitat use. Combining GPS acquired movement data may solve this issue and further enhance the winter ecology understanding of target species.

For this reason, to explore the wintering ecology of the osprey *Pandion haliaetus*, our target species, we relied on both GPS tracking and stable multi-isotopes of carbon, nitrogen and sulphur. The osprey is the only piscivorous diurnal raptor, of necessity living in strict association with aquatic environments (Cramp and Simmons 1980). In the Mediterranean basin, breeding sites are mostly located on the rocky cliffs of the islands and in coastal wetlands (Monti et al. 2020). GPS tracking studies revealed that Mediterranean ospreys are partial migrants and differ from their conspecifics belonging to northern central European populations, with respect to both temporal (total migration speed and use of stopovers) and spatial (distance, routes and main direction) components of migration strategy (Monti et al. 2018a). The great majority of Mediterranean ospreys wintered within the Mediterranean basin, spending the winter in southern Spain, Mediterranean islands and northern Africa. In some cases, some individuals behave as residents, staying at breeding grounds all year round (Monti et al. 2018b). Only a few individuals from the Mediterranean region winter in sub-Saharan Africa, as their conspecifics from northern Europe commonly do (Monti et al. 2018a).

In this study, we attempted to understand the range and feeding habitat of Mediterranean ospreys during winter using stable isotopes analyses (SIA) of feathers for a larger sample of individuals than is available from satellite tracked birds, to infer winter range and habitat use at the population level. A secondary aim of the study was to test whether SIA was a reliable tool for inferring wintering ground locations and habitat types used during the non-breeding period without deploying additional telemetry tags (since the costs of GPS devices is high, potentially limiting the sample size of tracked individuals). Because ospreys generally moult their body and flight feathers in winter (Prevost 1983; Electronic Supplementary Material S1), we expect that the isotopic chemical composition of the feathers reflects that of the habitat used in winter. Therefore, differences in stable isotopic ratios present in feathers would help determining if individuals wintered at different latitudes and foraged in different habitats (e.g., Bearhop et al. 1999; Inger and Bearhop 2008). To assess the isotopic method we used the GPS tracking data of ospreys’ movements as a reference for migratory routes and wintering sites’ location and habitat, allowing calibration of the isotopic composition with wintering locations.

Therefore, we expect these isotopic ratio values in osprey feathers to be potential proxies (a) to investigate latitudinal variation (Kelly et al. 2002; Farmer et al. 2004) and locate ospreys’ wintering grounds at a broad geographical scale; and (b) to link aquatic habitat types to different foraging areas in winter (Hobson 1999; Romanek et al. 2000).

## Materials and methods

### Study species

The osprey is a medium-size raptor species tightly linked to aquatic habitats, with a cosmopolitan distribution (Poole 2019). In the Western Palearctic, its breeding range stretches from Arctic countries like Finland and Norway to temperate Europe, where it breeds close to freshwater rivers and lakes (Cramp and Simmons 1980). At lower latitudes, as in the Mediterranean islands and North African coasts, as well as in Canary and Cape Verde archipelago, the osprey breeds in marine environments and coastal wetlands (Siverio et al. 2018; Monti et al. 2018a). Wintering birds also live close to marine environments along the Atlantic coast of Africa. Depending on latitude, reproduction generally starts in March–April; eggs hatch in April–May and chicks fledge in June-July. Birds depart in migration in August across a large front, eventually crossing the Mediterranean Sea (Duriez et al. 2018). They remain at wintering sites from October to February, where they moult their flight feathers (Prevost 1983). Just before spring migration (February), ospreys generally stop moulting after having undergone a complete or nearly complete moult of the primaries (Prevost 1983—Electronic Supplementary Material S1).

### GPS tracking

Wintering ecology was assessed for 12 adult ospreys from the Mediterranean population, including 5 birds from Balearic Islands and 7 from Corsica (Electronic Supplementary Material S2). Five adults were trapped during both summer and winter seasons in the Albufera wetlands (Mallorca Island, Balearics), using a perch-trap (e.g., Prevost 1984). These birds were fitted with 30-gr Solar Argos/GPS PTT-100 s (Microwave Telemetry Inc., USA). An additional seven adults (five females and two males) were caught along the western coast of Corsica, France, before the onset of the breeding season in March–April 2013, using a noose carpet laid on the nest. These individuals were equipped with a 24-g solar powered GPS/GSM device (model Duck-4, Ecotone, Poland). These seven Corsican individuals were also sampled for stable isotopes analyses (see below), and used as a validation sample.

All tags were programmed to collect data at hourly intervals during the whole winter season. For migratory birds, the wintering season was defined as the period between the arrival on the wintering grounds after the post-breeding migration and the onset of the next pre-breeding migration (following Strandberg et al. 2008; Monti et al. 2018b). For resident birds, winter was considered as spanning from October to February, according to the biology of osprey at these latitudes (Poole 1989; Monti et al. 2018b). GPS locations were imported into QGIS (v. 3.6.1) and projected to the Universal Transverse Mercator (UTM) coordinate system for all spatial analyses.

Seven birds were tracked for more than 2 consecutive years, providing a total of 20 wintering events (Electronic Supplementary Material S2). However, to avoid any pseudo-replication bias during analyses, for individuals from Balearics, we systematically selected only the most recent wintering event. This approach was the most conservative to maximize the chance to record an adult non-breeding home range instead of an immature (exact birds’ age at ringing was unknown). For Corsican adult birds, we considered the first wintering event that was the temporally closest when feathers have been sampled for isotopes analyses. Our dataset was hence composed of 12 individual wintering events. All GPS tracking data can be consulted in the Movebank data-repository (www.movebank.org; project study name: “Osprey in the Mediterranean”).

To describe the non-breeding areas, we estimated the individuals’ home ranges (95% kernel) and core areas (50% kernel) based on all winter GPS locations through fixed kernel density contours (sensu Worton 1989), using the Home Range App (https://shiny.cefe.cnrs.fr/HRApp/) through the R “*adehabitatHR*” package (Calenge 2011). Since ospreys are strictly associated with the presence of water bodies to catch fish (Cramp and Simmons 1980), we qualified habitat type composition during winter only according to aquatic environments: we calculated the percentage of marine water, brackish water or freshwater within the home range for each wintering event separately (see Monti et al. 2018b). This was achieved using satellite-images from Google Earth and available data in the literature as a source, together with local surveys, when feasible.

### Stable isotope ecobiogeochemistry

#### Control sample

**Fig. 1:**
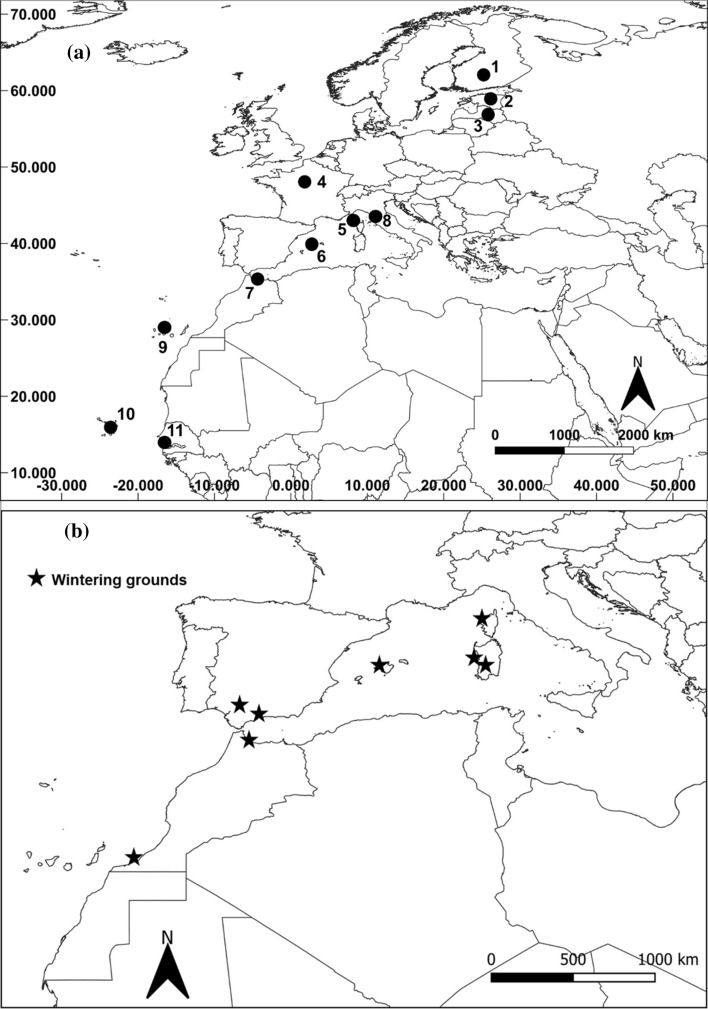
Maps with location of sites used for (**a**) feathers sampling (feathers of the control sample—black dots and numbers that refer to countries are reported in Table 1) and (**b**) wintering grounds of GPS tagged ospreys (black stars; see Electronic Supplementary Material S2)

To have a baseline resolution of the isotopic variation over a large latitudinal gradient and between different habitat types within the osprey’s breeding range, we collected feathers from a control sample. Overall, we collected feathers belonging to 75 different individuals (65 nestlings and 10 adults), from 11 sites throughout the Western Palearctic: from Arctic Finland to Morocco and West Africa (Cape Verde islands). The nests sampled were located near three main aquatic habitats: (a) freshwater; (b) brackish water and (c) marine water (Table 1; Fig. 1a).

**Table 1:** Distribution of sample size according to latitude and aquatic habitat. Sample size may differ for δ^13^C and δ^34^S analysis (see methods)

Country	Latitude	Habitat	Age	N_sampled individuals	δ^13^C samples	δ^34^S samples
(1) Finland	64° 00′ N	Freshwater	Chicks	10	10	1
(2) Estonia	59° 00′ N	Freshwater	chicks	4	4	1
(3) Latvia	57° 00′N	Freshwater	Chicks	5	5	1
(4) France	46° 00′ N	Freshwater	Chicks	11	11	3
(5) Corsica	42° 05′ N	?	Adults	18	18	18
	42° 05′ N	Marine water	Chicks	12	12	5
(6) Balearic Islands	39° 40′ N	Marine water	Chicks	7	7	3
(7) Morocco	35° 00′N	Marine water	Chicks	7	7	2
(8) Italy	42° 50′ N	Brackish water	Chicks	3	3	3
(9) Canary Islands	28° 15′ N	Marine water	Chicks	4	4	3
(10) Cape Verde Islands	16° 00′ N	Marine water	Adults	7	7	2
	16° 00′ N	Marine water	Chicks	2	2	0
(11) Senegal	14° 00′ N	Brackish water	Adults	3	3	3
Total				93	93	45

Numbers in brackets correspond to the site locations in Fig. 1

Chicks’ feathers were collected during ringing actions at the nest in summer, to measure the isotopic values of the environment around breeding sites during breeding period. More specifically, we sampled body feathers of 30 chicks from Continental Europe (Finland, Estonia, Latvia and France; Table 1, Fig. 1a), where ospreys nest close to freshwater sites and chicks are mostly fed with stenohaline fish species (Poole 1989). We also sampled the body feathers of 29 chicks from the Mediterranean basin (Table 1): 26 from 3 osprey populations breeding on rocky pinnacles at sea (Corsica, Morocco and Balearics Islands), feeding on euryhaline species (Francour and Thibault 1996; Thibault et al. 2001) and 3 chick samples from coastal wetlands (e.g., in Italy, where chicks are fed with marine, brackish or freshwater fishes; Monti et al. 2020). We also included an additional six samples from chicks on Atlantic islands (Canary and Cape Verde islands) to have other marine isotopic signatures from lower latitudes in the Atlantic, to be compared with those of the Mediterranean area. We also added samples from adult ospreys wintering in West Africa (Senegal), to have a signature of wintering grounds used by northern European ospreys (Alerstam et al. 2006; Bai and Schmidt 2011). In this case, we collected a piece (about 2 cm length) of vexillum at the tip of primaries (actively moulted) from three specimens of adult osprey belonging to the museum collection of the IRD (Institut de la Recherche et du Développement of Dakar, Senegal; Table 1; Fig. 1a).

#### Experimental sample

For inferring whether adults breeding in Corsica use wintering grounds different from breeding areas, we sampled 18 adult ospreys trapped in March 2012 and 2013 at their nests in Corsica, before the onset of the breeding season (7 of these were also fitted with GPS loggers; see above). From these birds, we selected only recently moulted feathers (darker in colour and with more regular borders; Electronic Supplementary Material S1). For the analyses, we used only the first 2 cm of the tip of two primaries and two secondaries that had grown during the previous winter and, thus, contained the isotopic signature of wintering grounds (Electronic Supplementary Material S1).

### Stable isotope analyses

Feathers belonging to 93 different individuals (75 control sample + 18 experimental sample) were analysed for stable isotopic ratios of Carbon (δ^13^C) and Nitrogen (δ^15^N) and 45 for Sulphur isotopic ratios (δ^34^S) (Table 1; Electronic Supplementary Material S1, S3 and S6). Sample size differs between C, N and S isotopic ratios because of limited quantity of feathers for certain individuals. Data for Nitrogen (*N* = 93) were not included in mixing models, as the number of isotopes should not be greater than *n*-1, *n* being the number of sources (see below; following Parnell et al. 2010). Moreover, while the osprey is a highly specialized piscivore, it is an opportunistic forager that can feed on a large variety of fish from a large range of trophic levels (Poole 1989), thus we expected large overlaps of Nitrogen values, even between different aquatic habitats (Electronic Supplementary Material S3 and S5). For these reasons, nitrogen isotopic results are not presented here but provided in Electronic Supplementary Material S3.

Feathers were thoroughly washed in distilled water using an ultra-sonic bath and then dried in an oven at 50 °C over night (Guillemain et al. 2014). Fragments of appropriate weight of feathers (for *C* = 0.3 mg, for *S* = 1 mg) were cut and placed in 8 × 5 mm EuroVector tin capsules. Carbon analysis of the Western Palearctic samples were conducted at the isotope platform of ISEM by means of a mass spectrometer Micromass Optima-AC117-coupled to an elemental analyser EuroVector 3000. Carbon analysis of the Western African samples were conducted at the isotopic platform LIENSs of University of La Rochelle using the isotope ratio mass spectrometer in continuous flow (CF-IRMS) Delta V Advantage, coupled with a Flash EA 1112 elemental analyser.

Stable isotope ratios were reported as deviations from a standard in per mil (‰) using the δ notation:

$$ \delta^{m} X = 1000*\left( {{{{\text{R}}_{{{\text{sample}}}} } \mathord{\left/ {\vphantom {{{\text{R}}_{{{\text{sample}}}} } {{\text{R}}_{{{\text{standards}}}} }}} \right. \kern-\nulldelimiterspace} {{\text{R}}_{{{\text{standards}}}} }} - 1} \right) $$

where *δ* is the isotope ratio of the sample relative to a standard, (i.e., ^13^C, ^34^S), R_sample_ and *R*_*standard*_ are the fractions of heavy to light isotopes in the sample and standard, respectively (i.e., ^13^C/^12^C, ^34^S/^33^S).

The precision for C isotopic ratios is higher than 0.1‰ for both ISEM and LIENSs mass spectrometer devices. The C isotope of the alanine standards of the ISEM laboratory was measured on both ISEM and LIENSs machines showing no difference (−23.7‰). All the sulphur analyses were run at LIENSs laboratory. The precision was higher than 0.3‰.

As a preliminary methodological test, we analysed 30 feathers from 15 individuals (15 primaries and 15 secondaries) to assess whether isotopic signatures of C and N vary in relation to the type of feather (Zelanko et al. 2011). Because we found no significant differences in isotopic signatures of C and N between primary and secondary feather (Wilcoxon signed-rank test: δ^13^C: *p* = 0.24; δ^15^N: *p* = 0.40; *N* = 15), we used both feathers types for our analyses.

### Statistical analysis

We first tested the hypothesis that feathers of control sample displayed significantly different isotopic signatures between the three habitats (freshwater, brackish water and marine water). Kruskall–Wallis tests were performed to analyse separately the differences in δ^13^C and δ^34^S ratios across habitats. A Dunn post hoc test was conducted when a significant difference was observed. To better discriminate habitat signatures, we performed permutational-MANOVA (PERMANOVA, adonis function in R) with 1000 permutations and using a Euclidean distance similarity index.

A common application of mixing models (Parnell et al. 2010) is to use the stable isotopic composition of individuals (usually called “consumers” in SIA) and their different type of foods (usually called “sources” in SIA) to make inferences about the composition of the animal’s assimilated diet. Here, we applied a mixing model to use isotopic signatures of Corsican adult ospreys (consumers) and ospreys from different putative habitats (sources) to infer wintering habitat of the former. Bayesian mixing models to solve relationships between organisms from stable isotope ratios models could show a bias toward a null generalist hypothesis when source estimates are inaccurate (Brett 2014), or when consumers fall outside the polygon bounding the proposed sources (i.e., when consumer isotopic values are not included in the range explained by the sources; Smith et al. 2013). We were particularly interested in the variance between age classes of Corsican ospreys because if a portion of the population is migratory, the range of isotopic values would increase the variance of that population. We used Student–Fischer test of equality of variance to compare values of Corsican adult ospreys and Corsican chicks and then, because the variance was not equal, we compared means with a Kruskall–Wallis test.

For application of the mixing model, samples from putative habitat were considered “sources”, and Corsican adult ospreys were considered “consumers”. As aforementioned, mixing models were firstly developed for inferring consumer diet from different sources considering uncertainty and trophic enrichment. There are some conceptual issues in the application of these tools to discriminate between habitats (Phillips et al. 2014). In particular, while a mixing model infers the diet of a consumer that can feed on multiple sources, this assumption could be wrong for habitat of Corsican ospreys visiting only one type of habitat. Following Taylor et al. (2016) and to identify consumers (adult Corsican ospreys) with isotopic values that were unlikely to be explained by the mixing model, we ran a stable isotope mixing polygon simulation (Smith et al. 2013) with 1500 iterations on the dataset. Corsican adult ospreys falling outside the resource polygon and having therefore a weak probability to be accurately assigned to a habitat were excluded from subsequent analyses (Electronic Supplementary Material S4). We ran a Bayesian mixing model (package *simmr* in R) with trophic enrichment factor set to zero. We assessed algorithm convergence using a Gelmann Diag diagnostic and checked the fit of the model with a posterior predictive check. Finally, to assess the reliability of the stable isotope analysis, we compared the habitat assigned by the mixing model with the habitats as observed for the seven individuals from Corsica that were also tracked by GPS and for which both winter location and habitat were exactly known.

## Results

### Differences in isotopic ratios between habitats

**Fig. 2:**
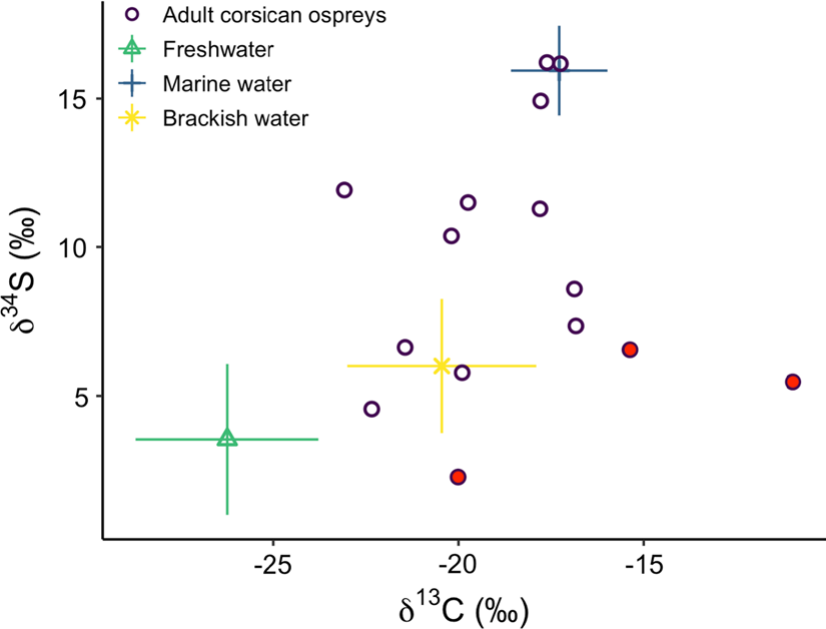
Isospace showing isotopic signatures (mean ± SE) of the three putative habitats (freshwater, marine water and brackish water) of the 18 adult Corsican ospreys (the 3 red points are adults falling outside the polygon bounding the habitat sources that were excluded from mixed model)

**Fig. 3:**
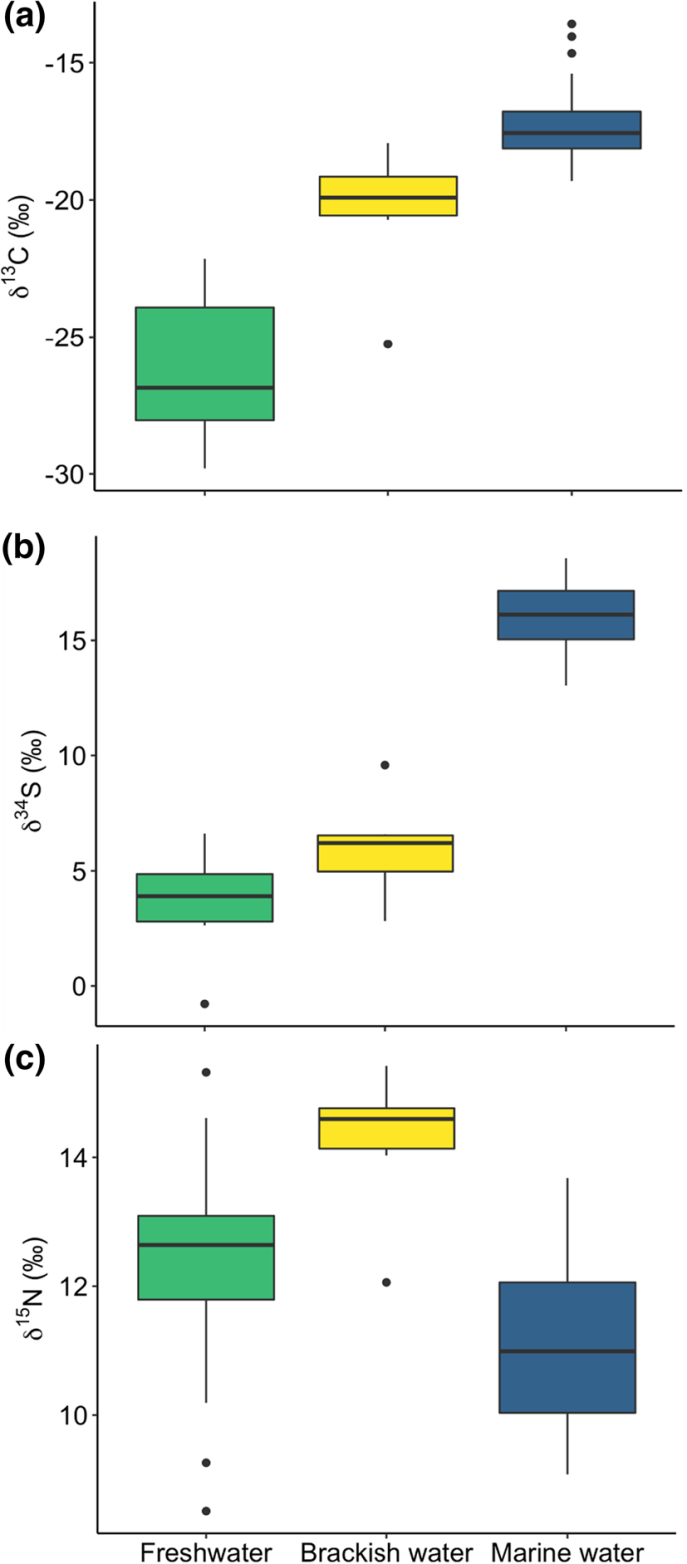
Differences in isotopic ratio for (**a**) δ^13^C, (**b**) δ^34^S and (**c**) δ^15^N in control samples over the three habitats: freshwater (green), brackish water (yellow) and marine water (blue)

Carbon and Sulphur isotopic ratios significantly differed between habitats (δ^13^C: *χ*^2^ = 56.79, *p* < 0.001, df = 2; δ^34^S: *χ*^2^ = 19.71, *p* < 0.001, df = 2; Figs. 2, 3 and Electronic Supplementary Material S5). Dunn post hoc test results revealed that δ^13^C and δ^34^S values increased with salinity, being higher in marine habitat than in brackish (δ^13^C: *Z* =  − 2.28, *p* = 0.022; δ^34^S: *Z* =  − 3.13, *p* < 0.01), and freshwater habitats (δ^13^C: *Z* =  − 7.52, *p* < 0.001; δ^34^S: *Z* =  − 3.91, *p* < 0.001). Although isotope values were not significantly different between brackish and freshwater habitat (δ^13^C: *Z* = 1.84, *p* = 0.065; δ^34^S: *Z* = 0.65, *p* = 0.51; Fig. 2). However, PERMANOVA analysis revealed that the combination of the two isotopes differed significantly among habitats (*F* = 187.92, *p* < 0.001, df = 2). Moreover, pairwise PERMANOVA showed difference among all possible pairs of the three habitats (marine water vs freshwater: *F* = 402.81, *p* < 0.01, df = 1; marine water v*s* brackish water: *F* = 76.25, *p* < 0.01, df = 1; freshwater *vs* brackish water: *F* = 22.78, *p* < 0.01, df = 1).

**Fig. 4:**
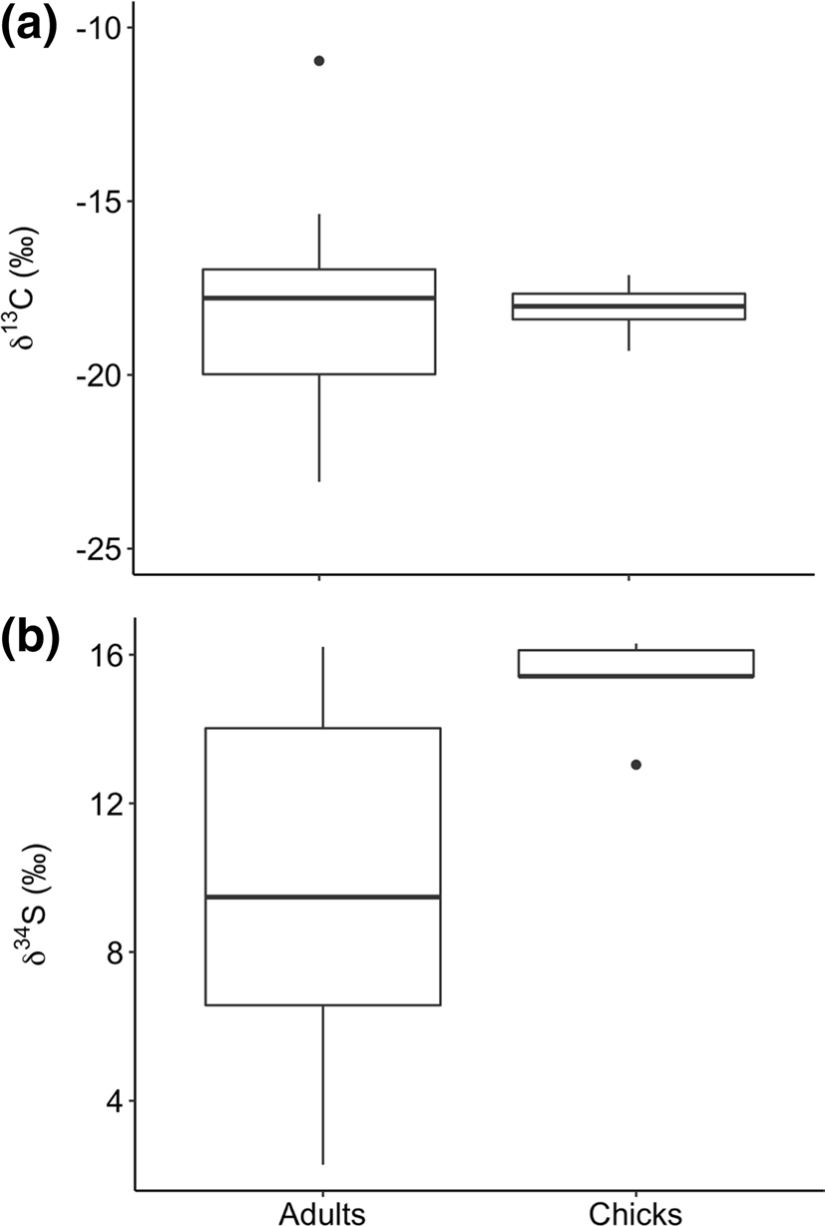
**a** δ^13^C and (**b**) δ^34^S mean values in feathers of both Corsican adults and chicks ospreys

Mean isotopic values for Corsican adults were significantly different from chicks for δ^34^S (*t*_1_ = 6.42, *p* < 0.001) but not for δ^13^C (*t*_1_ = 0.03, *p* = 0.857) (Fig. 4). Moreover, the variance between the two isotopes was higher in adults than in chicks (δ^13^C: *t*_17_ = 15.87, *p* < 0.001; δ^34^S: *t*_17_ = 11.04, *p* = 0.0316; Fig. 4).

### GPS tracking: movements and habitat selection

Ten out of 12 adult birds tracked with GPS spent the winter at temperate latitudes. Their wintering grounds ranged between 28°N and 42°N latitude within the Mediterranean basin (e.g., Spain, Morocco, Algeria, Sardinia-Corsica complex, Balearics and Italy). The only two individuals that crossed the Sahara desert reached the Atlantic coast of southern Morocco and the Senegal River delta in Mauritania in 2009 (Electronic Supplementary Material S2). 50% of tagged individuals were residents (six individuals) and 50% (six individuals) migrated. Migratory ospreys spent about 6 months (mean: 172.7 ± 25.2 days, *N* = 6; Electronic Supplementary Material S2) at wintering grounds.

Adult ospreys generally used a unique site throughout the whole winter. Daily movements were restricted (mean = 12.11 ± 6.8 km per day) and exploratory movements were only occasional (e.g., an individual covered 140 km, coming back to its main wintering site on the same day). Home range sizes were very small during wintering period (home range = 58.25 ± 42.9 km^2^; core area = 7.25 ± 3.8 km^2^; Electronic Supplementary Material S2) and did not differ between resident and migratory birds (Mann–Whitney *U* Test: home range: *U* = 12, *p* = 0.37, *N* = 12; core areas: *U* = 14, *p* = 0.57, *N* = 12).

The birds tracked during consecutive wintering seasons showed a high inter-annual site-fidelity, as most of them used the same areas that they had frequented in the previous years (mean overlap home ranges: 41.8 ± 20.2%; *N* = 7; Electronic Supplementary Material S2).

In winter, adult ospreys used coastal marine water habitats (bays and coastal waters), brackish water habitats (estuaries) as well as freshwater sites in both coastal and inland areas (marshes, dams and artificial ponds). Inter-individual plasticity in habitat choice (calculated on 95% home ranges) was high, with 8.3% of the birds using only freshwater sites and 33.3% using exclusively brackish habitats such as marshland or coastal lagoons. The remaining 58.33% frequented different habitats during the same season (Table 1 and Electronic Supplementary Material S5). No bird has had a home range fully associated with the marine environment, but those who used different habitats have frequented marine and coastal areas.

### Wintering habitats of experimental Corsican ospreys

To infer wintering location, the seven adults tracked by GPS, for which the exact wintering ground and habitat was known, were used as a validation sample. Stable isotope mixing polygon simulation showed that three consumers had isotopic values that were unlikely to be explained by the model and therefore a model solution was possible for 15 out of 18 (83%) of the Corsican adult ospreys. Thus, the remaining three (all not GPS-tagged birds) were excluded from Bayesian analysis (Electronic Supplementary Material S4).

**Fig. 5:**
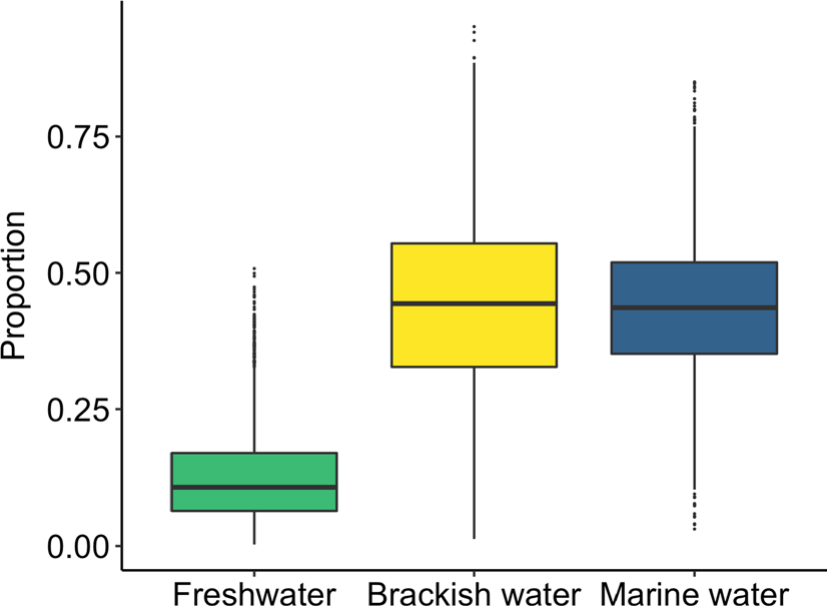
Predicted contribution of the three supplied sources (freshwater in green, brackish water in yellow and marine water in blue) for Corsican adult osprey’s isotopic signature (*n* = 15)

**Fig. 6:**
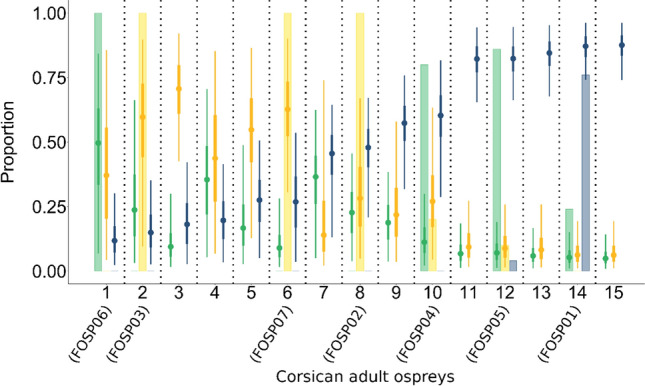
Predicted proportion for the contribution of the three supplied sources (freshwater in green, brackish water in yellow and marine water in blue) for each Corsican adult osprey isotopic signature. Reported values are the lower and upper 50% and 95% Bayesian credibility intervals predicted by the mixing models. For the seven individuals from Corsica that were also tracked by GPS (for whom precise wintering location and habitat composition is known) coloured bars represent the percentage of each habitat used from GPS tracks. ID of the GPS code is reported between brackets (see Electronic Supplementary Material S2)

For the remaining 15 Corsican adult ospreys, the mixing model identified marine water as the most frequented habitat, followed by brackish water and freshwater (Fig. 5). Each adult Corsican osprey revealed that the isotopic signature was characteristic of marine water, brackish water, or intermediate values between the two, but never close to freshwater isotopic values (Fig. 6). Five individuals exhibited typically marine isotopic signatures (birds 11–15 in Fig. 6; 95% Bayesian credibility intervals for estimated marine water contribution were higher than 65% and did not overlap other source contribution intervals), while one individual displayed isotopic signatures characteristic of a bird feeding almost only in brackish water (bird 3 in Fig. 6). Three other individuals fed mostly in marine habitat (birds 8–10 in Fig. 6) and three mostly in brackish water habitat (birds 2, 5 and 6 in Fig. 6). The remaining three Corsican adult ospreys (birds 1, 4 and 7) fed in variable combinations of the three habitats and dietary proportions were quite uncertain.

## Discussion

We performed a multi-isotopic analysis to determine the isotopic ratios and improve resolution in assessing osprey wintering ecology. Results from a control sample showed that the stable isotope ratios of osprey feathers varied greatly between different breeding habitats and latitudes in the Western Palearctic and West Africa. Significant differences in isotopic ratio efficiently allowed distinguishing chicks fed and reared in freshwater, brackish or marine environments over the latitudinal gradient. Results were concordant with the ecology of osprey breeding populations (populations nesting in northern Europe being more reliant on freshwater habitats while Mediterranean populations relying mostly on marine or brackish water habitats; Monti et al. 2020).

These trends allow the comparison of isotopic ratios in Corsican adult ospreys feathers with those of Corsican chicks fed in a marine habitat in the Mediterranean. The direct comparison of aquatic habitat composition available in the home range and the habitat predicted from SIA discriminant analysis for seven adult ospreys with known wintering sites revealed an overall good match between the two techniques (Fig. 6). The differences of habitats predicted by SIA and home range for two–three birds might be the result of a bird feeding in one habitat (e.g., seashore) but roosting in trees near freshwater. This would put freshwater habitat in the bird’s winter home range, even though it was not foraging in fresh water. Hourly GPS fixes are likely to miss foraging trips conducted between 2 consecutive hourly fixes. For example, a bird could be perched in a freshwater habitat at 1 hourly fix, fly out at close coastal wetlands or at sea, catch a fish, and be back to its perch before the next hourly fix (e.g., Electronic Supplementary Material S2). In addition, one should remember that the SIA was applied to feathers collected at the time of tagging the bird (i.e., moulted in the winter before the capture), while the home range analysis was performed in the winter after the capture. Thus, this calibration analysis was strongly relying on the assumption that adult ospreys are very faithful to their wintering area in subsequent years, as showed in Monti et al. (2018b) for the Mediterranean population, and as assessed in other populations across species’ distribution range (Washburn et al. 2014; Poole 2019). However, we cannot exclude the possibility that some birds may have used different wintering grounds before and after the capture, resulting in a mismatch between the two methods. Overall SIA discriminant analysis seems to be a powerful method to determine habitat used in winter for ospreys (and possibly other migratory waterbirds) that were not tracked by GPS.

The combined use of SIA and GPS tracking confirmed the partial migratory behaviour of the Mediterranean population. However, a potential bias in our study could be related to the fact that we used some birds trapped in winter in the Balearics (see methods); that might have increased the likelihood of capturing sedentary individuals, since migratory ones may have already left at that time of capture. The adult Corsican ospreys, captured in spring before reproduction, are probably more representative of the proportion of sedentary and migratory birds than the Balearic sample.

Nonetheless, we recorded a high plasticity in wintering strategies of Mediterranean ospreys. Even if it were impossible to determine exactly the latitude of each wintering area, SIA revealed that the majority of Corsican ospreys overwinter in a habitat different from their breeding habitat. Only 33% (5 out of 15 individuals) of Corsican adult feathers had isotopic values similar to those of Corsican chicks (marine habitat), suggesting a similar proportion of residents in this population. This resident behaviour in the Mediterranean osprey population was confirmed by GPS tracking (50% of tagged birds did not migrate). The remaining individuals showing different isotopic values could suggests three potential outcomes: (1) adults get all of their isotope sources from local habitats on the wintering grounds—in that case these adults had most likely spent the winter in different habitats (such as brackish and freshwater sites) located away from Corsica, because such sites are rare in Corsica (but more common in the neighbouring Sardinia for example) and are rarely, if ever, used by ospreys in winter (Thibault et al. 1996); (2) the adults actually use different isotopic sources, that stored from fat and other tissues (capital) as well as that from their diet (income), to grow their feathers, thus differing from that of the chicks; (3) there might be some differences in how growing chicks metabolically discriminate between heavy and light isotopes relative to the adults.

Furthermore, low variances in δ^13^C and in δ^34^S within each breeding area suggested that the source of organic matter in the food web was similar for all sampled chicks (e.g., marine prey during breeding season). This is in accordance with the strictly piscivorous habits of the species and the limited movements recorded during the breeding period (Monti et al. 2018c). Then, despite the small sample size, the lack of a significant difference in isotopic ratios between adults (mean δ^13^C: − 15.1 ± 1.1; mean δ^15^N: 12.4 ± 0.5; *N* = 7) and chicks (mean δ^13^C: −16.2 ± 0.6; mean δ^15^N: 11.5 ± 0.6; *N* = 2) from the Cape Verde islands also suggests that adults in this population are mostly resident, as reported in the literature (Poole 1989). However, this cannot be a conclusive evidence that chicks and resident adults have similar isotopic signatures owing to feeding on similar resources, especially because a test of the difference cannot be applied in this case.

Mediterranean ospreys tracked in winter mostly used temperate areas, but different habitat types (from marine bays to marshlands and, to a lesser extent, freshwater sites). While on one hand a high inter-individual variability was detected in the habitat used in winter within the Mediterranean population, on the other hand, each adult individual tended to use only one site (rarely two). This general lack of mobility in winter supported and indeed strengthened our assumptions made with SIA. Once arrived at the wintering ground, adult birds rarely moved around, but rather exploited a small area associated with a specific water body (Monti et al. 2018b).

This study proved that the integration of multiple SIA and tracking techniques can be useful to overcome the intrinsic limits of each method and achieve greater information about the basic ecology of migratory birds. The main limitation of SIA was the correlation between latitude of the sampling site and habitat type for carbon that prevented us from discerning the exact latitude of wintering grounds. For example, δ^13^C and δ^34^S values for samples from Senegal were equivalent to those recorded for Italy, where ospreys fish mostly in brackish water habitats in both regions. The downside of GPS tracking is obviously its cost, limiting the sample size, but compensated by a high resolution to determine wintering sites and movements in winter. This study showed that using SIA together with GPS tracking provides strong insights on the trophic level and shifts in diet composition in migratory birds wintering in aquatic habitats and where both stenohaline and euryhaline prey species can be present.

## Supplementary Information

Below is the link to the electronic supplementary material.

Supplementary file1 (PDF 1662 KB)
