# Supplementary material for: Using GPS tracking and stable multi-isotopes for estimating habitat use and winter range in Palearctic ospreys
Source: Oecologia. 2021 Jan 21;195(3):655–66. doi: 10.1007/s00442-021-04855-5 (PMC7940332; doi:10.1007/s00442-021-04855-5)
Supplement: Supplementary file 1 — Supplementary file1 (PDF 1662 KB) [file 442_2021_4855_MOESM1_ESM.pdf]

Article title: **Using GPS tracking and stable multi-isotopes for estimating habitat use and winter range in Palearctic ospreys**

Journal name: **Oecologia**

Author names:

Flavio Monti[1*$], Aloïs Robert[2$], Jean-Marie Dominici[3], Andrea Sforzi[4], Rafel Triay Bagur[5], Antoni Muñoz Navarro[6], Gaël Guillou[7], Olivier Duriez[2] and Ilham Bentaleb[8].

*Corresponding author: flaviomonti00@gmail.com

[1]University of Siena, Department of Physical Sciences, Earth and Environment, Via Mattioli 4, 53100 Siena, Italy

ORCID ID: https://orcid.org/0000-0001-8835-1021

[$]Flavio Monti and Aloïs Robert contributed equally to this work.

Electronic Supplementary Material

S1-Osprey moult scheme

Adult ospreys have an irregular moult sequence of the primary feathers, which occurs in successive waves (Figure S1), each starting at inner primary 1 and moving outwards to primary 10 (descendant) (Prevost 1983). If interrupted in one season, it resumes the next year from the points where it left off. Moult of the secondaries progresses towards the body (ascendant) and is completed after 17-19 months (Cramp and Simmons 1980). Moult mainly occurs from June-July to August-September and from October-November to February-March, resulting in an interruption during migratory periods (Prevost 1983).

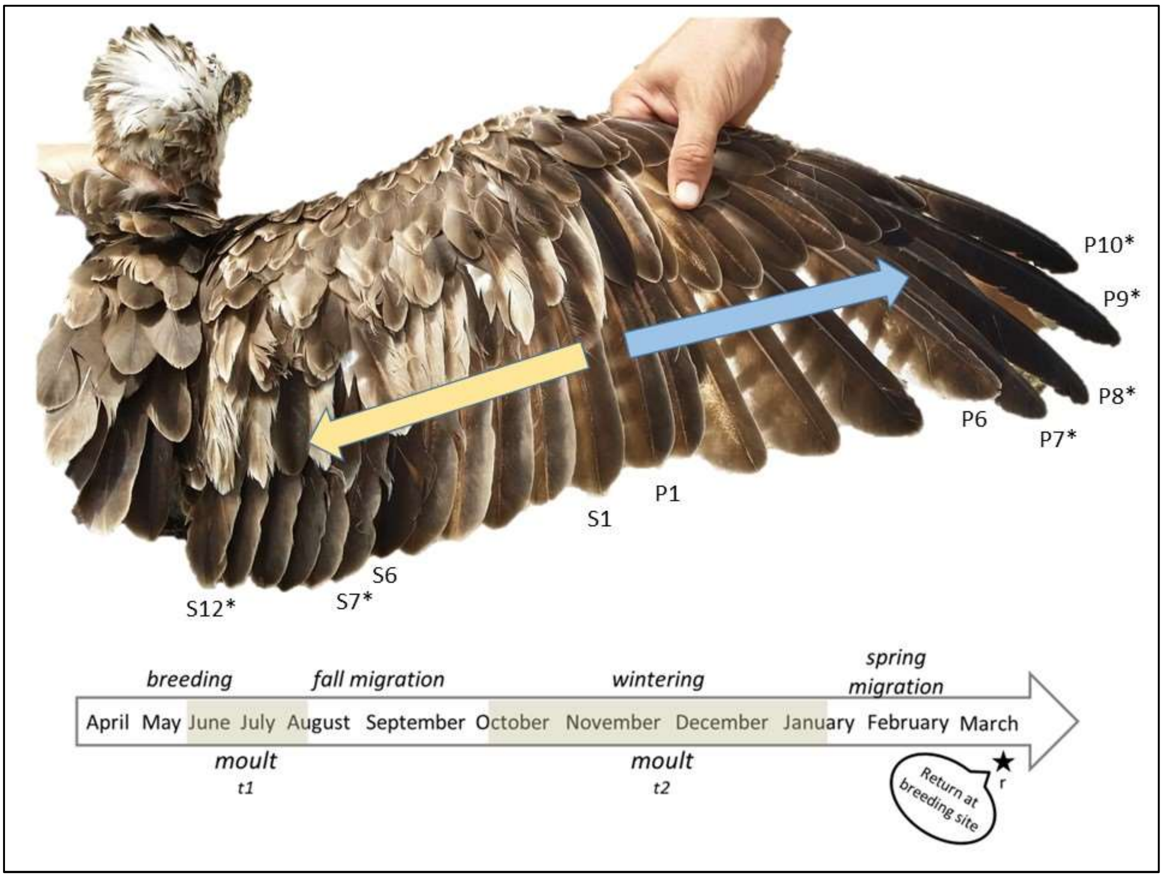

Figure S1: Osprey wing and moult pattern: blue arrow shows descendent moult for primaries and yellow arrow shows ascendant moult for secondaries. Recently moulted feathers are darker and sharper, and indicated with star. The phenology of ospreys and moult periods during the year is shown below: a feather moulted during breeding season (*t1*) will appear partially worn at time *r* (when birds return at breeding sites) and thus will not be selected for sampling; conversely a feather moulted in winter (*t2*) will appear as newly formed at time *r* and thus selected for sampling. In this case, we would collect a 2cm sample at the end of P7 and P8.

S2-Table of winter home ranges (fixed kernel 95%), core areas (fixed kernel 50%) and mean cumulative distances of daily movements of Mediterranean adult ospreys. Arrivals, departures and time spent (days) at wintering sites is reported for migratory individuals. For resident birds, the winter period has been considered between October and February (so time elapsed between arrival and departure dates is NA; see methods and Monti et al. 2018b). Country of wintering grounds is reported as well as the percentage of habitat type of home ranges for each wintering event. ID stands for tagging reference of each bird. * indicates individuals tracked for several years in which wintering season was not selected for analysis (only the most recent wintering was considered; see methods).

ID	Breeding origin	Winter	Arrival date	Departure date	Duration of tracking (days)	Duration of wintering period (days[1])	Core area (km^2)	Home range (km^2)	Home range overlap between years	Daily Distance (km/day)	Wintering country	Marine water %	Brackish water %	Fresh water %
FOSP01	Corsica	2013-2014	Oct	Feb	122	NA [1]	5.56	28.16	NA	NA [1]	Corsica	75.6	0	24.4
FOSP02	Corsica	2013-2014	05-oct	25-mar	171	171	2.92	15.74	NA	NA [1]	Morocco	0	100	0
FOSP03	Corsica	2013-2014	21-aug	05-jan	137	NA [2]	9.09	49.8	NA	10.76 ± 15.15	Sardinia	0	100	0
FOSP04	Corsica	2013-2014	15-sep	16-mar	182	182	3.45	25.98	48.8	7.8 ± 8.3	Spain	19	0	81
	Corsica	2014-2015	12-sep	07-dec	86	NA [1]	3.92	24.78		10.4 ± 5.3	Spain	25.2	0	74.8
FOSP05	Corsica	2013-2014	25-jul	06-feb	226	226	18.22	159.63	45.2	22.8 ± 32.5	Sardinia	3.8	10.5	85.7
	Corsica	2014-2015	30-jul	13-jan	197a	112	12.27	151.45		27.5 ± 28.4	Sardinia	8.8	15.6	75.6
FOSP06	Corsica	2013-2014	15-aug	19-mar	216	216	4.22	18.93	46.8	4.3 ± 8.3	Spain	0	0	100
	Corsica	2014-2015	24-aug	04-mar	192	192	4.44	23.18		7.4 ± 8.3	Spain	0	0	100
FOSP08	Corsica	2013-2014	17-aug	21-feb	188	188	6.88	58.31	34.6	12.7 ± 9.11	Morocco	0	100	0
	Corsica	2014-2015	22-aug	15-feb	177	177	7.39	30.88		9.6 ± 4.3	Morocco	0	100	0
BAL1M	Balearics	2010-2011	Oct	Feb	122	NA [3]	8.75	65.09	NA	11.48 ± 11.8	Balearics	35.4	64.6	0
BAL2F	Balearics	2009-2010	Oct	Feb	122	NA [3]	7.5	79.91	NA	3.35 ± 4.7	Balearics	57.2	42.8	0
BAL3IND	Balearics*	2010-2011	Oct	Feb	122	NA [3]	3.4	22.93	68.16	7.54 ± 8.3	Balearics	0.4	99.6	0
	Balearics*	2011-2012	Oct	Feb	122	NA	4.01	49.11		13.15 ± 18.7	Balearics	43	57	0
	Balearics	2012-2013	Oct	Feb	122	NA	2.73	18.75		6.94 ± 14.0	Balearics	0	100	0
BAL4M	Balearics*	2009-2010	Oct	Feb	122	NA[3]	13.74	121.57	49.62	22.17 ± 21.9	Balearics	26.8	73.2	0
	Balearics	2010-2011	Oct	Feb	122	NA	12.96	123.42		23.8 ± 20.7	Balearics	39.5	60.5	0

BAL5M	Balearics*	2009-2010	17-nov	25-mar	128	128	4.14	47.82	0	8.67 ± 6.8	Mauritania	91.1	8.9	0
	Balearics	2010-2011	Oct	Feb	122	NA	8.13	74.14		15.4 ± 13.9	Balearics	51.9	48.1	0
**Mean**						*172.75 ± 25.2*	*7.25 ± 3.8*	*58.25 ± 42.9*	*41.88 ± 20.9*	*12.11 ± 6.8*		*26.31 ± 28.4*	*50.11 ± 41.2*	*23.58 ± 38.7*

NA[1] : not applicable due to tags' failure before the end of the winter; NA[2] : incomplete season due to bird death; NA[3] : incomplete season because bird has been trapped in winter.

Figure S2: Examples of winter home ranges and core areas (white and green line, respectively) are reported for four individuals: a) FOSP01 wintering in the western coast of Corsica, b) FOSP05 wintering in the western coast of Sardinia (1st winter), c) FOSP06 wintering in southern Spain (1st winter) and d) BAL5M wintering in Mauritania (1st winter). Aquatic habitats (freshwater, brackish water and marine) have been reported on the maps.

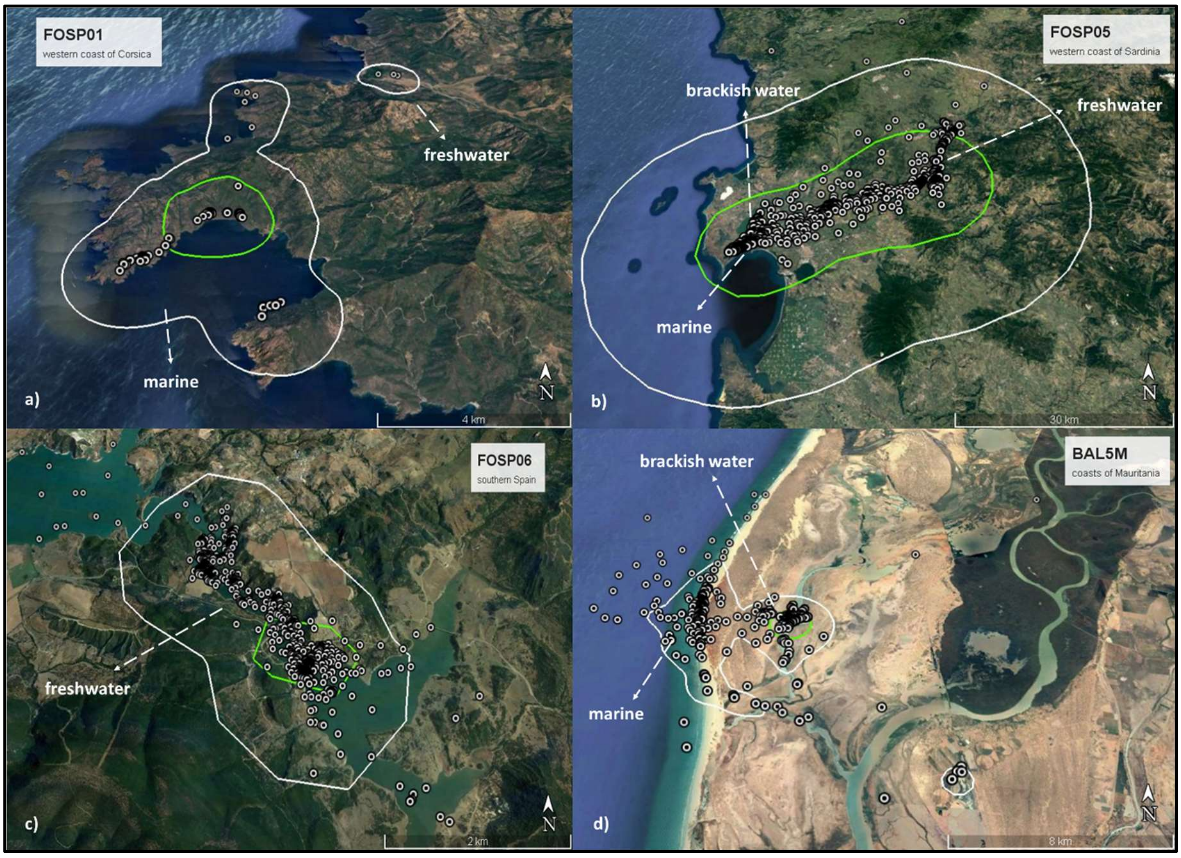

S3-Isotopic ratios of nitrogen $\delta^{15}N$

The isotopic ratios of Nitrogen ($\delta^{15}N$) are used as good indicators of foraging ecology patterns (Hobson 1999). As for the $\delta^{13}C$ (Bentaleb et al. 1998), the $\delta^{15}N$ of primary producers vary predictably among ocean basins, with high-latitude pelagic ecosystems having much lower $\delta^{15}N$ than lower latitudes (Cherel and Hobson 2007). A total of 93 feather samples belonging to different individuals was analysed for stable isotopes of Nitrogen ($\delta^{15}N$) (Tab. a). Fragments of appropriate weight of feathers (for N = 0.3 mg) were cut and placed in 8x5 mm EuroVector tin capsules. Nitrogen of Palearctic samples was analysed at the isotope platform of Institut des Sciences de l'Evolution de Montpellier (France) by means of a mass spectrometer Micromass Optima-AC117-coupled to an elemental analyser EuroVector 3000. Nitrogen of samples collected in tropical feathers was analysed at the isotopic platform LIENSs of University of La Rochelle using the isotope ratio mass spectrometer in continuous flow (CF-IRMS) Delta V Advantage, coupled with a Flash EA 1112 elemental analyser. The precision for N isotopic ratios is higher than 0.1 ‰ for both ISEM and LIENSs mass spectrometer devices. The N (-0.5‰) isotope of the alanine standards of the ISEM laboratory was measured on both ISEM and LIENSs machines showing a significant difference for Nitrogen of +0.34‰ at the LIENSs. The results have been corrected considering this value.

As we did for the carbon, we analysed 30 feathers of 15 individuals (15 primaries and 15 secondaries) to assess whether isotopic signatures of N vary in relation to the type of feather (Zelanko et al. 2011). Because we found no significant differences in isotopic signatures of N between primary and secondary feathers (Wilcoxon signed-rank test: $\delta^{15}N$: $p = 0.4$), we used both feathers types for our analyses.

We used One-way ANOVA to analyse the variance of $\delta^{15}N$ across latitude (i.e. sampling sites). We started from the null hypothesis that isotopic ratios in feathers of Corsican adult ospreys were not significantly different from those of Corsican chicks (supporting a residency hypothesis). Even if we tested for differences in the mean, our main interest was in the variance between age classes because if a portion of the population is migratory, the range of isotopic values would increase the variance of that population. We used Student-Fischer test of equality of variance to compare values of Corsican adult ospreys and Corsican chicks and then, because the variance was not equal, we compared means with a Kruskall-Wallis test. Correlation was shown by Pearson correlation test.

Table S3: Distribution of sample size according to latitude and aquatic habitat for δ^{15}N. Numbers in brackets correspond to the site locations in Fig. 1.

Country	Latitude	Habitat	Age	N_sampled individuals	δ^{15}N samples
(1) Finland	64° 00' N	Freshwater	chicks	10	10
(2) Estonia	59° 00' N	Freshwater	chicks	4	4
(3) Latvia	57° 00'N	Freshwater	chicks	5	5
(4) France	46° 00' N	Freshwater	chicks	11	11
(5) Corsica	42° 05' N	?	adults	18	18
		Marine water	chicks	12	12
(6) Balearic Islands	39° 40' N	Marine water	chicks	7	7
(7) Morocco	35° 00'N	Marine water	chicks	7	7
(8) Italy	42° 50' N	Brackish water	chicks	3	3
(9) Canary Islands	28° 15' N	Marine water	chicks	4	4
(10) Cape Verde Islands	16° 00' N	Marine water	adults	7	7
			chicks	2	2
(11) Senegal	14° 00' N	Brackish water	adults	3	3
**Total**				93	93

Main results

Values of the N isotope found in osprey feathers (used as control) were different between breeding sites across the Western Palearctic and tropical Africa (δ^{15}N: t_{10} = 6.61, p <0.001) (Fig.3 and Fig. S3a). However, no pattern associated with a particular aquatic habitat was noticeable for δ^{15}N (t_{10} = 6.61, p <0.001), which varied greatly from one site to another and within each site regardless of its latitude. Interestingly, in Cape Verde, where we sampled both adults (n=7) (known to be mostly sedentary) and chicks (n=2), values were equivalent in both age classes, even if sample size was not sufficient to perform statistical tests (n = 7: mean δ^{15}N = 12.46 ± 0.55 ‰ in adults *vs* 11.53 ± 0.63 ‰ in chicks). Values of δ^{15}N were highly correlated to δ^{34}S (p <0.001, r^2 = -0.72). Mean isotopic values for Corsican adults were significantly different from chicks for δ^{15}N (t1 = 13.57 ± 3.03 ‰, p <0.001), with the variance higher in adults than in chicks (δ^{15}N: t17 = 9.99 ‰ ± 0.78, p <0.001; Fig. S3b).

Figure S3a: Variations of $\delta^{15}N$ isotopic ratios of osprey feathers (used as control) over the latitudinal gradient in the Western Palearctic and West Africa (skewed bars = continental Europe, grey bars = Mediterranean, white bars = Atlantic islands and black bars = Senegal). Numbers refer to countries reported in Tab. S3. Finland has been split into south (1) and north (1*) for a better visual inspection.

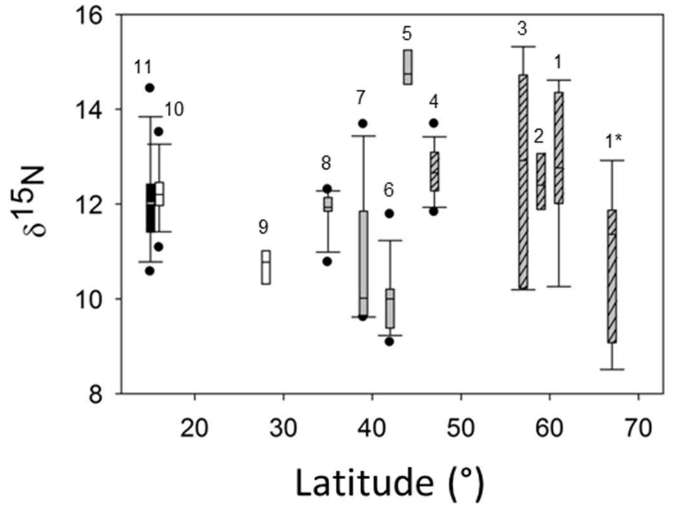

Figure S3b: Difference in isotopic ratios of $\delta^{15}N$ in feathers of both Corsican adults and chicks ospreys.

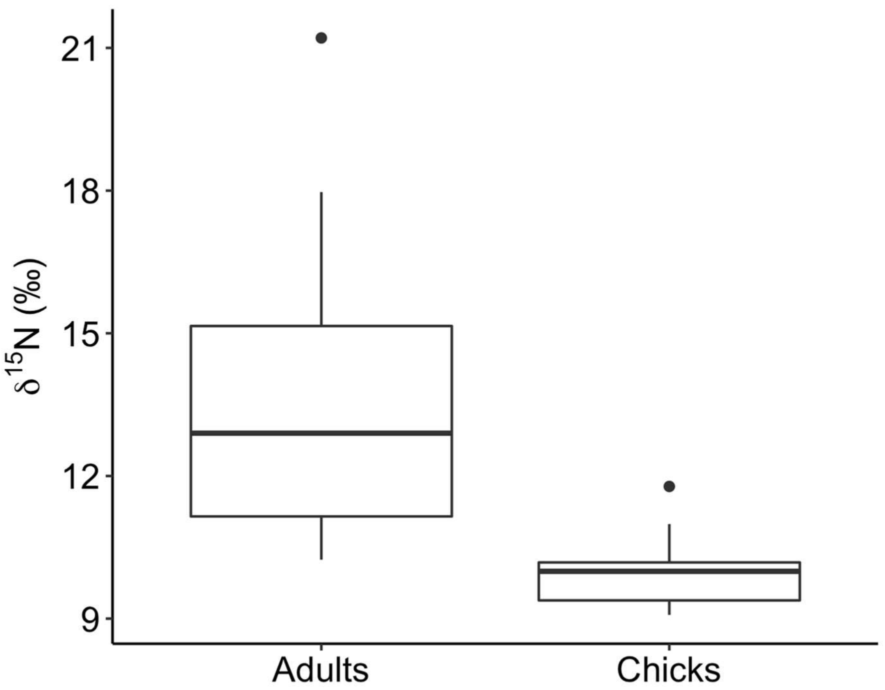

S4-Stable isotope mixing polygon simulation

Figure S4: The simulated mixing region for the 18 Corsican adults. The positions of the consumers (black dots) and the average source signatures (white crosses) are shown. Probability contours are at the 5% level (outermost contour) and at every 10% level. The 3 black dots falling outside of the probability contours represent those having a weak probability of being accurately assigned to a habitat and thus were excluded from subsequent analyses (see main text).

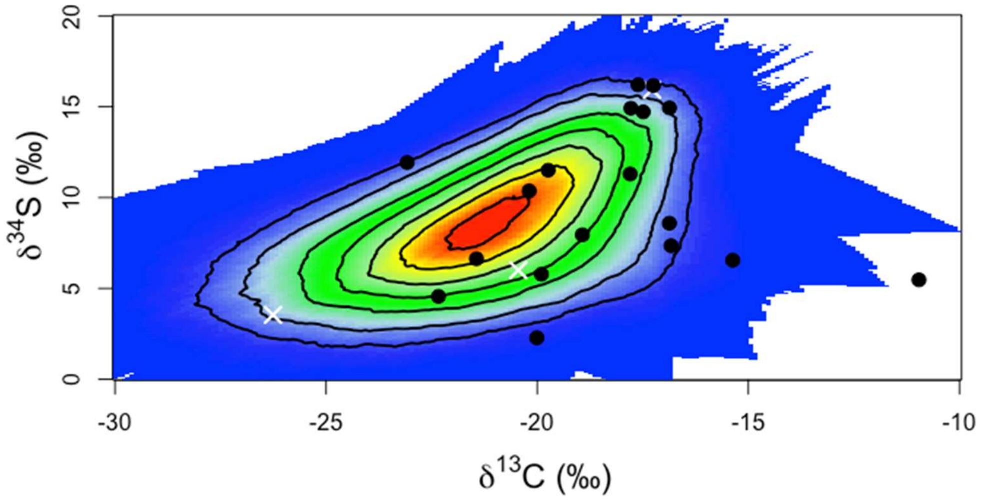

S5-Isotopic values between habitats

C, S and N isotopic values (mean ± SD) and number of individuals for the control sample of each of the three habitats. To refer also to Figure 2.

	**$\delta^{13}C$**		**$\delta^{34}S$**		**$\delta^{15}N$**	
**Habitat**	***N***	***Mean ± SD***	***N***	***Mean ± SD***	***N***	***Mean ± SD***
Freshwater	30	-26.26 ± 2.47	6	3.54 ± 2.53	30	12.36 ± 1.54
Brackish water	6	-20.45 ± 2.55	6	6.01 ± 2.25	6	14.25 ± 1.16
Marine water	39	-17.28 ± 1.30	15	15.94 ± 1.51	39	11.05 ± 1.26

S6- Nitrogen ($\delta^{15}N$), Carbon ($\delta^{13}C$) and Sulphur ($\delta^{34}S$) isotopic values in feathers from 93 different ospreys (75 control sample + 18 experimental sample) and between different aquatic habitat types within the osprey breeding range in the Western Palearctic.

ID	δ^{15}N	δ^{13}C	δ^{34}S	Site	Aquatic Habitat	Age class
Control1	9.44	-17.84	NA	Corsica	Marine water	chick
Control2	10.16	-18.13	15.41	Corsica	Marine water	chick
Control3	9.39	-18.38	15.42	Corsica	Marine water	chick
Control4	9.37	-17.77	16.30	Corsica	Marine water	chick
Control5	11.78	-19.31	NA	Corsica	Marine water	chick
Control6	9.89	-17.35	NA	Corsica	Marine water	chick
Control7	9.29	-18.86	NA	Corsica	Marine water	chick
Control8	10.25	-18.27	NA	Corsica	Marine water	chick
Control9	10.13	-17.13	13.04	Corsica	Marine water	chick
Control10	10.10	-17.13	16.12	Corsica	Marine water	chick
Control11	9.08	-18.47	NA	Corsica	Marine water	chick
Control12	10.99	-17.92	NA	Corsica	Marine water	chick
Control13	11.93	-16.72	16.09	Morocco	Marine water	chick
Control14	10.77	-17.28	16.40	Morocco	Marine water	chick
Control15	11.83	-18.54	NA	Morocco	Marine water	chick
Control16	12.30	-18.17	17.13	Morocco	Marine water	chick
Control17	12.20	-18.12	NA	Morocco	Marine water	chick
Control18	11.94	-17.68	NA	Morocco	Marine water	chick
Control19	11.90	-17.53	NA	Morocco	Marine water	chick
Control20	10.06	-17.54	17.34	Canary Islands	Marine water	chick
Control21	10.98	-16.85	NA	Canary Islands	Marine water	chick
Control22	10.56	-17.64	17.18	Canary Islands	Marine water	chick
Control23	11.04	-17.56	14.23	Canary Islands	Marine water	chick
Control24	11.08	-15.76	NA	Cape Verde	Marine water	chick
Control25	11.98	-16.55	NA	Cape Verde	Marine water	chick
Control26	12.20	-16.12	18.57	Cape Verde	Marine water	adult
Control27	13.51	-15.40	NA	Cape Verde	Marine water	adult
Control28	12.89	-13.59	17.28	Cape Verde	Marine water	adult
Control29	12.14	-15.58	NA	Cape Verde	Marine water	adult
Control30	12.27	-14.66	NA	Cape Verde	Marine water	adult
Control31	11.92	-14.05	NA	Cape Verde	Marine water	adult
Control32	12.31	-16.70	NA	Cape Verde	Marine water	adult
Control33	10.19	-29.80	NA	Latvia	Freshwater	chick
Control34	10.24	-29.31	NA	Latvia	Freshwater	chick
Control35	15.32	-28.36	NA	Latvia	Freshwater	chick
Control36	14.52	-27.81	2.63	Latvia	Freshwater	chick
Control37	12.92	-24.07	NA	Latvia	Freshwater	chick
Control38	13.33	-29.61	NA	Estonia	Freshwater	chick
Control39	11.99	-27.08	NA	Estonia	Freshwater	chick
Control40	11.78	-26.76	NA	Estonia	Freshwater	chick

Control41	12.80	-29.50	6.62	Estonia	Freshwater	chick
Control42	14.61	-23.55	-0.78	Finland	Freshwater	chick
Control43	14.27	-23.53	NA	Finland	Freshwater	chick
Control44	12.59	-27.24	NA	Finland	Freshwater	chick
Control45	12.76	-27.55	NA	Finland	Freshwater	chick
Control46	10.26	-29.74	NA	Finland	Freshwater	chick
Control47	12.92	-29.04	NA	Finland	Freshwater	chick
Control48	11.52	-27.89	NA	Finland	Freshwater	chick
Control49	11.36	-28.10	NA	Finland	Freshwater	chick
Control50	9.26	-24.05	NA	Finland	Freshwater	chick
Control51	8.51	-22.62	NA	Finland	Freshwater	chick
Control52	11.83	-25.92	NA	France	Freshwater	chick
Control53	12.96	-25.11	NA	France	Freshwater	chick
Control54	13.69	-25.36	3.3	France	Freshwater	chick
Control55	11.99	-26.97	NA	France	Freshwater	chick
Control56	12.19	-23.11	4.5	France	Freshwater	chick
Control57	12.98	-23.28	NA	France	Freshwater	chick
Control58	12.55	-22.42	NA	France	Freshwater	chick
Control59	12.66	-22.14	4.98	France	Freshwater	chick
Control60	12.62	-23.87	NA	France	Freshwater	chick
Control61	13.13	-27.21	NA	France	Freshwater	chick
Control62	13.24	-26.73	NA	France	Freshwater	chick
Control63	15.42	-20.1	6.03	Italy	Brackish water	chick
Control64	14.74	-19.72	6.39	Italy	Brackish water	chick
Control65	14.45	-17.93	6.59	Italy	Brackish water	chick
Control66	10.01	-17.39	NA	Balearic Islands	Marine water	chick
Control67	9.70	-16.84	14.68	Balearic Islands	Marine water	chick
Control68	9.63	-17.73	NA	Balearic Islands	Marine water	chick
Control69	10.10	-17.6	NA	Balearic Islands	Marine water	chick
Control70	9.61	-17.89	NA	Balearic Islands	Marine water	chick
Control71	13.68	-19.2	NA	Balearic Islands	Marine water	chick
Control72	12.43	-18.7	13.87	Balearic Islands	Marine water	chick
Control73	14.77	-20.72	4.62	Senegal	Brackish water	adult
Control74	12.06	-18.96	9.58	Senegal	Brackish water	adult
Control75	14.03	-25.27	2.82	Senegal	Brackish water	adult
Exp1	-23.08	21.21	11.93	Corsica	?	adult
Exp2	-20.19	16.28	10.37	Corsica	?	adult
Exp3	-19.90	17.32	5.78	Corsica	?	adult
Exp4	-17.80	12.62	11.3	Corsica	?	adult
Exp5	-17.61	11.02	16.21	Corsica	?	adult
Exp6	-17.78	10.56	14.92	Corsica	?	adult
Exp7	-22.34	13.18	4.56	Corsica	?	adult
Exp8	-16.87	12.25	8.59	Corsica	?	adult
Exp9	-21.44	17.97	6.63	Corsica	?	adult
Exp10	-16.83	11.11	7.35	Corsica	?	adult
Exp11	-20.01	13.61	2.28	Corsica	?	adult
Exp12	-15.37	13.95	6.55	Corsica	?	adult
Exp13	-19.74	15.49	11.51	Corsica	?	adult
Exp14	-17.25	10.79	16.17	Corsica	?	adult

Exp15	-10.96	14.14	5.47	Corsica	?	adult
Exp16	-18.93	11.27	7.95	Corsica	?	adult
Exp17	-16.87	11.31	14.94	Corsica	?	adult
Exp18	-17.49	10.24	14.72	Corsica	?	adult

References:

Cramp S, Simmons KEL (1980) Handbook of the birds of Europe, the Middle East and North Africa. The birds of the Western Palearctic, Vol. 2. Oxford University Press. Oxford.

Bentaleb I, Fontugne M, Descolas-Gros C, Girardin C, Mariotti A, Pierre C, Brunet C, Poisson A (1998) Carbon isotopic fractionation by plankton in the Southern Indian Ocean: relationship between $\delta^{13}C$ of particulate organic carbon and dissolved carbon dioxide. Journal of Marine Systems 17:39-58.

Cherel Y, Hobson KA (2007) Geographical variation in carbon stable isotope signatures of marine predators: a tool to investigate their foraging areas in the Southern Ocean. Marine Ecology Progress Series 329:281–287.

Hobson KA (1999) Tracing diets and origins of migratory birds using stable isotope techniques. - Society of Canadian Ornithologists Special Publication, Fredericton, New Brunswick: 21-41.

Monti F, Grémillet D, Sforzi A, Sammuri G, Dominici JM, Triay Bagur R, Muñoz Navarro A, Fusani L, Duriez O (2018b) Migration and wintering strategies in vulnerable Mediterranean Osprey populations. Ibis 160:554-567. doi:10.1111/ibi.12567

Prevost YA (1983) The moult of the Osprey. Ardea 71:199-209.

Zelanko PM, Rice NH, Velinsky DJ (2011) Using carbon and nitrogen stable isotopes to distinguish the locations of feather growth in osprey (*Pandion haliaetus*). Proc. Acad. Nat. Sci. Phila. 161:61-72.
